# Retinal Pigment Epithelium Transplantation: Past, Present, and Future

**DOI:** 10.18502/jovr.v17i4.12325

**Published:** 2022-11-29

**Authors:** Ayyad Zartasht Khan, Tor Paaske Utheim, Jon Roger Eidet

**Affiliations:** ^1^Department of Medical Biochemistry, Oslo University Hospital, Kirkeveien 166, Nydalen, Oslo, Norway; ^2^Department of Oral Biology, Faculty of Dentistry, University of Oslo, Sognsvannsveien 10, Oslo, Norway; ^3^Department of Plastic and Reconstructive Surgery, Oslo University Hospital, Kirkeveien 166, Nydalen, Oslo, Norway; ^4^Department of Ophthalmology, Sørlandet Hospital Arendal, Lundsiden, Kristiansand, Norway; ^5^Department of Ophthalmology, Stavanger University Hospital, Stavanger, Norway; ^6^Department of Ophthalmology, Oslo University Hospital, Kirkeveien 166, Nydalen, Oslo, Norway; ^8^https://orcid.org/0000-0002-2048-225X

**Keywords:** Retinal Pigment Epithelium, Retinal Pigment Epithelium Transplantation, Regenerative Medicine, Vitreoretinal Surgery, Tissue Engineering.

## Abstract

Retinal pigment epithelium (RPE) is a monolayer of cells situated between photoreceptors and the underlying choroid. It is essential for normal retinal function. Damaged RPE is associated with diseases such as age-related macular degeneration, Stargardt's macular dystrophy, and retinitis pigmentosa. RPE cells can easily be visualized *in vivo*, sustainable* in vitro*, and differentiated from stem cells with a relatively straightforward protocol. Due to these properties and the clinical significance of this epithelium in various retinal diseases, RPE transplantation as a treatment modality has gained considerable interest in the last decade. This paper presents the main techniques for RPE transplantation and discusses recent clinically relevant publications.

##  INTRODUCTION

Retinal pigment epithelium (RPE) is a cell layer sandwiched between photoreceptors apically and Bruch's membrane basally. RPE cells are cuboidal with apical microvilli gaping photoreceptor outer segments.^[1]^ Their cytoplasms contain melanin pigments, lipofuscin granules, melanolipofuscins, and phagosomes.^[1]^ These cells adhere to each other via zonula occludentes, creating a mosaic-patterned layer of tightly adjoined hexagonally shaped cells. RPE performs several functions essential for vision, such as adsorption of excessive light, transport of nutrients to and from the neuroretina, protection against photooxidation, regeneration of 11 cis-retinal for the visual cycle, and phagocytosis of shed photoreceptor outer segments.^[1,2]^ It also constitutes the outer part of the blood–retinal barrier.^[1]^

RPE dysfunction is seen in several retinal disorders, such as age-related macular degeneration (AMD),^[3]^ proliferative vitreoretinopathy (PVR), and retinitis pigmentosa (RP). Regarding AMD, it is still unclear whether drusen formation is a consequence or a cause of RPE dysfunction.^[4]^ However, both are strongly associated with each other.^[5]^ In PVR, RPE cells contribute to the folding of the retina by detaching and translocating from the underlying basement membrane.^[6,7,8]^ Similarly, RPE disintegration and migration are responsible for the bone-spicule pigmentation that is pathognomonic for RP.^[9]^ Thus, when RPE fails to function correctly, the retina suffers.

Replacing RPE cells is an idea that arose shortly after the characterization of these cells about 40 years ago.^[10]^ Their anatomical accessibility, *in vitro* resilience, and clinical importance made them attractive for transplantation scientists. The first proof-of-concept study of RPE transplantation was performed in 1984 by Gouras et al in monkeys.^[11]^ Four years later, Li and Turner published a report demonstrating that subretinal injection of RPE cells prevented photoreceptor degeneration in rats.^[12]^ The field has subsequently matured at a rapid pace with not only animal studies,^[13,14,15]^ but also human trials, using RPE of fetal,^[16,17]^ *post-mortem* adult,^[18,19,20]^ autologous,^[21,22,23]^ induced pluripotent stem cell (iPSC)-derived^[24]^ and embryonic stem cell (ESC)-derived origin.^[25,26,27]^

The introduction of IPSC- and ESC-derived RPE have been critical for the advancement of clinical RPE research during the recent years, and has, to a large extent, solved the issue of sourcing RPE donor tissue. While iPSCs offer an unlimited supply of autologous cells and (to some degree) do not require the use of immunosuppressants, they may carry patients' own genetic vulnerabilities contributing to disease processes.^[28]^ This can be avoided by using ESCs. However, ESCs raise ethical concerns^[29,30,31]^ and are, in contrast to iPSCs, neither autologous nor unlimited in supply. The methods for generating RPE cells from iPSCs^[32,33]^ and ESCs^[34,35]^ have been described in detail. As the succeeding paragraphs will demonstrate, ongoing clinical trials employ these two stem cell sources to generate mature, transplantation-ready RPE.

### Transplantation Techniques

There are currently three techniques for subretinal RPE transplantation: (1) surgical placement of RPE as an intact cell sheet (with or without scaffold), (2) injection of RPE as a cell suspension, and (3) macular translocation.

Transplantation of RPE as an intact cell sheet was first described in 1991 by Peyman et al, who treated one patient with an autologous pedicle flap and another with an allogeneic graft consisting of RPE with an underlying choroid.^[19]^ Similar procedures have later been performed by others.^[24,26,36,37]^ Delivering RPE as a patch increases the likelihood of correct anatomical placement and the structural integrity of the graft. Major complications associated with this technique are subretinal hemorrhage and proliferative vitreoretinopathy. Delivery by injection is technically easier and probably less traumatizing to the adjacent tissue. However, cell clumping, poor attachment, and disorganization of RPE upon injection^[38]^ are important drawbacks. The third approach, macular translocation, involves rotating the retina away from a subretinal pathology to an area of healthy RPE. This technique is surgically less straightforward and complicated by cataract, retinal detachment, and diplopia.^[39]^

**Table 1:** RPE transplantation trials listed on ClinicalTrials.gov.


**No.**	**Study title**	**Status**	**Publications**	**Conditions**	**Sponsor/** **Collaborators**	**Phase**	**Study type**	**Estimated study start **	**Estimated study completion **	**Study location (country)**	**NCT number**	**Related projects (NCT numbers)**
**1**	Safety and Tolerability of RPESC-derived RPE Transplantation in Patients with dAMD	Not yet recruiting	No Publications Available	dAMD	Luxa Biotechnology, LLC; NIH; NEI; Regenerative Research Foundation	Phase 1/2	Interventional	February 2022	September 2025	USA	NCT04627428	
**2**	Treatment of RP and LCA by Primary RPE Transplantation	Unknown status	No Publications Available	LCA; RP	Eyecure Therapeutics Inc.; Beijing Tongren Hospital	Phase 1	Interventional	August 2018	March 2020	China	NCT03566147	
**3**	Autologous Transplantation of Induced Pluripotent Stem Cell-Derived RPE for Geographic Atrophy Associated with AMD	Recruiting	No Publications Available	AMD	NIH; NEI	Phase 1/2	Interventional	September 2020	May 2029	U.S.A.	NCT04339764	
**4**	Subretinal Transplantation of RPE in Treatment of AMD	Unknown status	No Publications Available	dAMD	Chinese Academy of Sciences; Beijing Tongren Hospital	Phase 1/2	Interventional	January 2018	December 2020	China	NCT02755428	NCT03944239
**5**	Treatment of dAMD with RPE Derived from Human Embryonic Stem Cells	Unknown status	No Publications Available	dAMD	Chinese Academy of Sciences; The First Affiliated Hospital of Zhengzhou University	Phase 1/2	Interventional	September 2017	December 2020	China	NCT03046407	
**6**	Clinical Study of Subretinal Transplantation of Human Embryo Stem Cell Derived RPE in Treatment of Macular Degeneration Diseases	Unknown status	No Publications Available	A SMD	Southwest Hospital, China	Phase 1/2	Interventional	May 2015	December 2019	China	NCT02749734	
**7**	Transplantation of Autologous RPE Versus Translocation of Autologous RPE and Choroid in AMD	Completed	^[42]^	AMD	The Ludwig Boltzmann Institute of Retinology and Biomicroscopic Laser Surgery	Not Applicable	Interventional	February 2004	September 2008	Austria	NCT00401713	
**8**	Safety and Tolerability of Sub-retinal Transplantation of Human Embryonic Stem Cell Derived RPE in Patients With SMD	Completed	^[27,43]^	SMD	Astellas Institute for Regenerative Medicine	Phase 1/2	Interventional	December 13, 2011	September 30, 2015	U.K.	NCT01469832	NCT02941991; NCT01345006; NCT01344993; NCT02445612; NCT02463344; NCT02563782; NCT02122159; NCT03167203
**9**	Study of Subretinal Implantation of Human Embryonic Stem Cell-Derived RPE Cells in Advanced dAMD	Active, not recruiting	^[40,41]^	dAMD	Regenerative Patch Technologies, LLC	Phase 1/2	Interventional	February 2016	June 2023	U.S.A.	
**10**	RPE Safety Study for Patients in B4711001	Active, not recruiting	^[26]^	AMD	Moorfields Eye Hospital NHS Foundation Trust	Phase 1/2	Interventional	September 2016	October 2020	U.K.	NCT03102138	
**11**	A Phase I/IIa, Open-Label, Single-Center, Prospective Study to Determine the Safety and Tolerability of Sub-retinal Transplantation of Human Embryonic Stem Cell Derived RPE (MA09-hRPE) in Patients with Advanced dAMD	Unknown status	No Publications Available	dAMD	CHABiotech CO., Ltd	Phase 1/2	Interventional	September 2012	June 2020	Republic of Korea	NCT01674829	NCT01625559; NCT03305029
**12**	Safety and Efficacy Study of OpRegen for Treatment of Advanced Dry-Form Age-Related Macular Degeneration	Active, not recruiting	No Publications Available	AMD	Lineage Cell Therapeutics, Inc.; CellCure Neurosciences Ltd.	Phase 1/2	Interventional	April 2015	December 2024	U.S.A.; Israel	NCT02286089	

white<bcol>13</ecol>NCT, National Clinical Trial; RPESC, retinal pigment epithelial stem cell; dAMD, dry age-related macular degeneration; NIH, National Institutes of Health; NEI, National Eye Institute; RP, retinitis pigmentosa; LCA, Leber congenital amaurosis; RPE, retinal pigment epithelium; AMD, age-related macular degeneration; SMD, Stargardt's macular dystrophy; MMD, myopic macular degeneration.

### Current State of the RPE Transplantation

To give the reader an idea of the current state of RPE transplantation, selected recent studies are briefly discussed below.

In a phase 1/2 clinical trial, Schwartz and associates injected human ESC-derived RPE subretinally via a small 38-gauge retinotomy in 18 patients; nine with AMD and nine with Stargardt's Macular Dystrophy (SMD).^[27]^ Visual acuity improved in the majority of patients. No ocular or systemic safety issues were registered, apart from surgery-associated complications, such as vitreous inflammation, cataract, and endophthalmitis. This study suggested that ESC-derived RPE is a potential and safe source of cells for the treatment of retinal disorders.

While Schwartz et al delivered the cells via injection, Kashani and colleagues described a patch of ESC-derived RPE monolayer attached to a synthetic parylene substrate.^[40]^ The initiative is called California Project to Cure Blindness. They implanted this engineered patch in 16 patients with advanced non-neovascular AMD with a median age of 78 years in a single-arm, open-label, prospective, non-randomized, phase 1/2 study.^[41]^ Critical inclusion criteria were advanced non-neovascular AMD, pseudophakia, and severe vision loss. Mild to moderate subretinal hemorrhages and macular holes were reported as adverse events. One patient developed ischemic colitis, possibly related to immunosuppression.

Employing a similar approach, an initiative called “The London Project to Cure Blindness” published data on a phase 1 trial encompassing two patients with severe wet AMD.^[26]^ Their patch was also made of differentiated ESC-derived RPE, but the scaffold was made of polyester membrane coated with human vitronectin. Using purpose-built microsurgical tools, the RPE patches were delivered subretinally to one eye in each of the two patients with severe exudative AMD. They reported successful delivery and survival of the RPE patch and a visual acuity gain of 29 and 21 letters in the two patients, respectively, over 12 months. Importantly, preclinical safety studies did not reveal tumorigenicity or notable proliferative capacity of the ESC-derived RPE cells. Undifferentiated ESCs were not detected in the final differentiated RPE product. Furthermore, investigation of systemic biodistribution 26 weeks after implantation of the RPE grafts in pigs did not provide any evidence of migration of cells distant to the administration site. Although the number of patients included in the study is too low to conclude on the clinical efficiency of the RPE patch, the report provides valuable information about the surgical technique, stability of the transplant, and the safety of the ESC-derived cells.

##  CONCLUSION

As the aforementioned reports indicate, human RPE transplantation is still in its early days. Significant challenges related to graft composition, graft vehicle, and surgical technique need to be addressed. However, it is encouraging that, despite these challenges, the mentioned studies report positive outcomes following transplantation. Future clinical trials are therefore eagerly awaited. As of this writing, 12 RPE transplantation projects are listed on the clinical trials database ClinicalTrials.gov [Table 1], all being phase 1/2 trials assessing safety, side effects, and dosing. The most frequently occurring indication remains AMD. However, SMD, RP, and Leber congenital amaurosis are other diseases listed as indications for some ongoing trials. The majority of the projects seem to employ RPE cells derived from ESC. Based on this and the aforementioned recent studies, it is the authors' impression that the current front-line of RPE transplantation is based on cell delivery as a patch using ESC-derived RPE.

##  Financial Support and Sponsorship

None.

##  Conflicts of Interest

There are no conflicts of interest.

TPU and JRE hold a patent on the culture of retinal pigment epithelial cells: https://patents.google.com/patent/US20180119097A1/en.
